# Propolis volatile compounds: chemical diversity and biological activity: a review

**DOI:** 10.1186/1752-153X-8-28

**Published:** 2014-05-02

**Authors:** Vassya Bankova, Milena Popova, Boryana Trusheva

**Affiliations:** 1 Institute of Organic Chemistry with Centre of Phytochemistry, Acad. G. Bonchev strl. bl. 9, 1113 Sofia, Bulgaria

**Keywords:** Propolis, Volatiles, Biological activity, Plant origin

## Abstract

Propolis is a sticky material collected by bees from plants, and used in the hive as building material and defensive substance. It has been popular as a remedy in Europe since ancient times. Nowadays, propolis use in over-the-counter preparations, “bio”-cosmetics and functional foods, etc., increases. Volatile compounds are found in low concentrations in propolis, but their aroma and significant biological activity make them important for propolis characterisation. Propolis is a plant-derived product: its chemical composition depends on the local flora at the site of collection, thus it offers a significant chemical diversity. The role of propolis volatiles in identification of its plant origin is discussed. The available data about chemical composition of propolis volatiles from different geographic regions are reviewed, demonstrating significant chemical variability. The contribution of volatiles and their constituents to the biological activities of propolis is considered. Future perspectives in research on propolis volatiles are outlined, especially in studying activities other than antimicrobial.

## Introduction

Bees have been in existence for over 100 mill years, and have become a perennial species that can exploit virtually all habitats in the world. This evolutionary success is due to their sophisticated social organization, but it is due also to the remarkable properties of the bee products. Honeybees produce high quality foods, building materials and unprecedented chemical weapons. Propolis is one of the most fascinating bee product, both building material and defensive substance. Bees make use of the mechanical properties of this resinous substance by applying it for blocking holes and cracks, repairing combs, strengthening the thin borders of the comb. On the other hand, they make use of its biological action: bee glue contains the putrefaction of “embalmed” intruders, killed in the hive and too large to be carried out, and it is responsible for the lower incidence of microorganisms within the hive than in the atmosphere outside [1]. The action against infections is an essential characteristic of propolis and this fact has been recognized and used by humans since ancient times. Modern science focused on propolis after the 1960’s, inspired by the general interest in natural products. In the last 50 years, numerous studies have revealed versatile biological activities of propolis: antibacterial, antifungal, antiviral, cytotoxic, antioxidant, anti-inflammatory, immunomodulatory among others [2-4]. Chemical studies were also performed, including studies on the essential oils and volatiles of propolis. It turned out that propolis composition is complex and very much variable in different regions [5]. Meanwhile, commercial interest to propolis is growing continuously, it is used as a component of food additives, cosmetics and over-the-counter preparations.

Propolis is a bee product of plant origin, thus at different geographic locations the source plants might vary with respect to the local flora. To produce propolis, bees collect vegetable material and mix it with wax. It is now generally accepted that bees collect resinous plant materials, produced by a variety of botanical processes, in different parts of plants: lipophilic materials on leaves and leaf buds, mucilages, gums, resins, latices, etc. [6]. In some cases, bees also may cut fragments of vegetative tissues to release the substances used in propolis production [7]. Of course, the specificity of the flora at the site of collection determines the chemical composition of propolis, including volatile compounds. In different ecosystems, bees collect propolis from different source plants, choosing appropriate representatives of the local flora. Thus, the term “propolis” does not have any specific chemical connotation unlike the scientific name of a plant species. Propolis contains secondary plant metabolites, including volatiles, but they are produced by different plant species and are not the same all over the world. In this overview we are going to focus on the volatiles of different propolis types according to their plant origin.

## Review

### The importance of propolis volatiles

Propolis volatiles give to the bee glue its specific pleasant aroma. It is well known that worker honeybees (*Apis mellifera*) respond to odors in several behavioral contexts [8,9]. Pheromones emitted by queens and/or workers communicate a variety of messages and elicit fairly stereotypical responses in the proper context. Workers also learn to respond to floral odors, which, prior to the learning experience, do not typically elicit such strong innate responses as do pheromones [10]. Workers learn the association of floral odors with the nectar and/or pollen rewards offered by the flowers, thus allowing for future identification of the flowers from which the rewards can be harvested [11]. No data exist about the role of odors in foraging for propolis so far. It seems only logical to assume that the same learning process occurs in the case of resin collection, taking into consideration the recent finding of Leonhardt et al., who have proved that stingless bees in Borneo use volatile terpenes as olfactory cues to find appropriate resin sources [12].

The volatile constituents of propolis play an important role also for human propolis users by contributing to its pleasant aroma and its biological activity.

### Chemical diversity of propolis volatile oils from different geographic regions

Many authors, in the introductions of their research articles on propolis claim that propolis contains up to 10% volatile oils, referring to data from articles published in the period 1908 – 1948 [1]. More recent publications however report a much lower percentage, usually up to 1%, rarely 2 - 3%. We have obtained similar results in our laboratory with samples from many different geographic regions.

#### *European propolis*

The chemical composition of propolis is crucial for understanding its biological activity. The first study of propolis essential oils, published in 1974, reported the identification of only a few constituents: benzoic acid, benzyl alcohol, vanillin, and eugenol [13]. Further studies revealed the variability of propolis volatile oils that seemed to be even higher than the variability of polar propolis constituents (such as phenolics compounds, flavonoids, phenolic acids, etc.). As propolis knowledge developed, it became clear that in the Temperate zone the basic plant source of bee glue are the bud exudates of trees of the genus *Populus*, mainly the black poplar *P. nigra*. Major constituents of propolis from the Temperate zone are the typical poplar phenolics: flavonoid aglycones and esters of substituted cinnamic acids [14]. The volatile oils however were more changeable in their chemical composition, especially with respect to the relative quantities of different constituents.

In most European propolis samples studied, sesquiterpenes predominate in the volatile oils (obtained by hydrodistillation, simultaneous hydrodistillation-extraction or headspace), followed by aromatic compounds, such as benzyl acetate, benzyl benzoate and benzyl alcohol (Table 1). β-Eudesmol was found to be the major constituent of propolis volatile oils from France, Hungary, Bulgaria and Northern Italy [15-18]. It is interesting to note that the same sesquiterpene alcohol was found to be the major constituent of essential oils distilled from leaf buds of *P. nigra *[19]. The poplar bud oil, just like propolis of the above mentioned origins, contained mainly sesquiterpenes.

**Table 1:** Propolis volatiles from different geographic origin

Method of isolation*	**Geographic origin**	**Percentage% (W/W)**	**Main constituents**	**Bioactivity**	**Reference**	**Year**
DE	Albania	0.3	cadinene (10.5%), methoxyacetophenone (9.0%), sesquiterpene alcohol M = 222 (18.5%)	NT**	[17]	1994
DE	Bulgaria	0.3	β-eudesmol (8.8%), δ-cadinen (5.3%), sesquiterpene alcohol M = 222 (15.5%)	Antibacterial	[17,20]	1994, 1999
DE	China (Inner Mongolia)	NA***	α –bisabolol (20.1%), 2-methyl-3-buten-2-ol (10.8%), 3-methyl-2-butene-1-ol (8.3%)	NT	[21]	2009
DE	Mongolia	0.6	benzyl benzoate (8.6%), Sesquiterpene alcohol M = 222 (15.7%)	NT	[17]	1994
DE	Brazil, stingless bees	0.1	*Tetragona*: nerolidol (12.3%), spatulenol (10.4%);	antibacterial	[22]	1999
			*Melipona quadrifasciata* – p-cimen-8-ol (11.4);			
			*M. comperittes –* ethylphenol (10.2%)			
DE	Mexico (Yucatan)	0.02	α-pinene (11.9%), hexadecanoic acid (10.9%), *trans-*verbenol (7.0%)	NT	[23]	2006
DE	Mexico (Yucatan), stingless bees	0.4	α-pinene (17.6%), β-caryophyllene (11.8%), spatulenol (9.7%), caryophyllene oxide (9.5%), β-bourbonene (9.2%)	NT	[23]	2006
DE	Canary islands	0.1-0.3	nerolidol (3.2 – 11.0%), spatulenol (3.2 – 8.4%), ledol (1.6 – 3.8%)	Antibacterial	[24]	2000
HD	Croatia	0.2	limonene (6.4 – 10.5%), benzyl alcohol (3.1 – 18.2%), benzyl benzoate (3.6 – 4.4%)	NT	[25]	1996
HD	Czech Republic	NA^*^	benzoic acid, benzyl alcohol, vanillin, eugenol	NT	[13]	1974
HD	Greece	0.03-0.1	α –pinene (7.9 – 45.8%), trans-β-terpineol (2.2 – 6.6%), Junipene (1.5 – 11.7%), δ-cadinene (0.3 – 8.4)	Antibacterial	[26]	2007
HD	Hungary	0.3–1.5	β-eudesmol, benzyl benzoate (2 types, one richer in benzyl benzoate)	Antibacterial	[16]	1988
HD	India (Maharashtra state)	3.2	tricosane (13.6%), hexacosane (11.5%), palmitic acid (8.5%), linalool (6.7%), methyleugenol (6.0%)	Repellent activity against the honeybee *Apis florea*	[27]	2013
HS, HD	Italy (Northern)	NA	benzoic acid (3.1 – 30.1%), benzyl benzoate (0.2 – 13.1%), β-eudesmol (2.9 – 12.9%), δ-cadinene (1.3 – 13.3%), γ-cadinene (1.4 – 8.9%), Т-cadinol (2.7 – 10.0%), α-cadinol (4.8 – 9.7%)	NT	[18]	2013
HD	Portugal	0.05	viridiflorol (9.0 – 39.0%), *n*-tricosane (5.3%), *n-*nonadecane (4.0 – 18.0%)	NT	[28]	2013
HD	China (Inner Mongolia)	NA	3-methyl-2-buten-1-ol (26.8%), phenylethyl alcohol (17.1%), 2-methoxy-4-vinylphenol (9.5%)	NT	[21]	2009
HD	Brazil	0.4	spatulenol (3.0 – 13.9%), (2Z,6E)-farnesol (1.6 – 14.9%), prenyl-acetophenone (0.2 – 8.7%), benzyl benzoate (0.3 – 18.3%)	NT	[29]	1998
HD	Brazil	0.1	β-caryophyllene (12.7%), acetophenone (12.3%)	Antibacterial	[30]	2010
HD	Brazil	NA	nerolidol (6.6%), trans-caryophyllene (4.1), spatulenol (3.6%)	NT	[31]	2008
HD	Brazil (Minas Gerais State)		(E)-nerolidol (17.1%), β-caryophyllene (13.4%), selina-3,7(11) diene (10.4%)	NT	[32]	2008
HD	Brazil (Teresina, Piaui State)	NA	1,8 – cineole (24.0%), exo-fenchol (11.3%), terpinen-4-ol (7.7%)	NT	[33]	2008
HD	Brazil (Piaui State)	NA	α-pinene (0.3 – 34.4%), E-caryophyllene (2.6 – 17.4%), α-copaene (3.6 – 7.5%)	NT	[33]	2008
HD	Brazil (Rio de Janeiro State)	0.04	α-pinene (18.3%), β-pinene (6.5%), δ-cadinene (7.0%)	Antifungal	[34]	2010
HD	Brazil (Rio Grande do Sul State)	3-3.8	α-pinene (57.0 – 63.0%), β-pinene (12.5 – 30.8%), limonene (1.5 – 11.2%)	Antibacterial	[35]	2012
HD	Ethiopia (Assela)	0.9	5,6,7,8-tetramethylbicyclo [4,1,0] hept-4-en-3-one (15.0%), acoradiene (13.8%), epicedrol (6.8%)	NT	[36]	2012
HD	Ethiopia (Haramaya)	1.2	calamenene (13.8%), 4-terpineol (8.6%), epi-bicyclosesquiphellandrene (8.4%)	NT	[36]	2012
SHS	Estonia	NA	eucalyptol (25.9%), α-pinene (20.6%), benzaldehyde (10.8%), β-pinene (8.9%)	NT	[37]	2014
SHS	China	NA	3-methyl-3-butene-1-ol (40.3%), 3-methyl-2-butene-1-ol (11.6%), 4-penten-1-yl acetate (9.0%), α-longipinene (9.4%)	NT	[37]	2014
SHS	Brazil	NA	α-pinene (52.5%), β-pinene (20.8%)	NT	[37]	2014
SHS	Uruguay	NA	α-pinene (23.0 – 53.4%), β –pinene (24.1 – 27.4%), limonene (2.1 – 15.6%)	NT	[37]	2014
HS	Italy (Southern)	NA	α-pinene (13.2%), germacrene D-4-ol (6.3%)	NT	[18]	2013
HS	UK (Wales)	NA	6-methylheptyl-5-en-2-one (16.0%), benzyl alcohol (14.2%), benzaldehyde (9.0%)	NT	[38]	1989
HS	Argentina (Andean region)	NA	*O-*cimene, limonene	NT	[39]	2011
DHS	China (Inner Mongolia)	NA	heptadecane (7.0%), phenantrene (4.0%)	NT	[21]	2009
DHS	China (Heilongjiang, Beijing)	NA	acetic acid (44.3 - 60.0%), benzyl alcohol (7.3 - 13.9%)	NT	[40]	2013
DHS	China (Shanghai)	NA	acetic acid (25.3%), cedrene (10.4%), 3-methyl-3-buten-1-ol (7.1%)	NT	[40]	2013
DHS	China (Shandong)	NA	acetic acid (11.4%), 1-(1,5-dimethyl-4-hexenyl)-4-methyl-benzene (9.7%), 1,2,3,4, 4a,5,6,8a-octahydro-4a,8-dimethyl-2-(1-methylethenyl)-naphthalene (8.3%), benzyl alcohol (7.4%)	NT	[40]	2013
HS-SPME	Turkey (Eastern Anatolia)	NA	phenyl ethyl alcohol (7.7%), benzyl alcohol (7.4%), decanal (6.7%), ethyl benzoate (6.5%)	Antimicrobial, antioxidant	[41]	2013
HS-SPME	Turkey (North Eastern Anatolia)	NA	cedrol (7.0 - 15.6%), α-bisabolol (14.3%), δ-cadinene (2.7 - 5.6%)	Antimicrobial, antioxidant	[41]	2013
HS-SPME	Turkey (South Eastern Anatolia)	NA	α –terpinene (21.8%), α –terpineol (12.3%), junipene (9.1%), cinnamyl alcohol (8.7%), β-cariophyllene (8.1%)	NT	[42]	2004
	Brazil, stingless *Friesomelita*	Bee legs samples	manool, totarol	NT	[43]	2002
Extraction	France	0.5	β-eudesmol (30.0%), guaiol (10.0%), benzyl benzoate (8.0%)	NT	[15]	1981
SD	Poland (Southern)	1.2 of ethanol extract	farnesol, dihydroeudesmol, guaiol	antibacterial	[44]	1983
UAE	Brazil	5.4 in raw 0.3 in ethanol	nerolidol (10.4 – 14.7%), benzenepropanoic acid (14.9 – 20.8%)	NT	[45]	2013
MAE	Brazil		longipinene (24.9%), α-eudesmol (6.9%), β-eudesmol (6.1%), β-caryophyllene (5.3%)	Therapeutic effect on anxiety	[46]	2012

*DE Distillation-extraction; *HD* hydrodestilation; *SHS* static headspace; *HS* headspace; *DHS* dynamic headspace; *HS-SPME* headspace solid-phase microextraction; *SD* steam destilation; *UAE* ultrasonic assisted extraction; *MAE* microwave assisted extraction. ** *NT* not tested; *** *NA* not available.

Of the non-terpenic compounds, benzyl alcohol and benzyl benzoate were found in these propolis samples. Benzyl benzoate was present in Hungarian samples, and also in many other samples from the temperate region [16,18]. Interestingly, benzyl benzoate was not detected in the volatile oils of poplar buds [19]. In general, it can be concluded that propolis of poplar origin has also volatile oils of poplar origin, at least in Hungary, Bulgaria, France and Northern Italy. Some of the observed differences could be due to chemical variations of the volatiles of different poplar subspecies and clones. It is known that the bud exudates even of the same species have demonstrated quantitative variability in a wide range.

In other regions of Europe, the chemical composition of the volatile oils showed some differences (Table 1). This could be due to the fact that other plant species could be playing an auxiliary role as propolis bearing plants: e.g. *Cupressus sempervirens* in Greece with major essential oil constituent α-pinene [47], as in the studied Greek propolis samples [26]. In propolis sample from Southern Italy (Adriatic coast) α-pinene was also identified in high percentage and coniferous species were suggested as the plant source [18]. Recently, monoterpenes α-pinene, β-pinene and eucalyptol in high amounts were detected in volatiles of Estonian propolis sample [37]. *Cistus ladanifer* and propolis samples [28] from the south regions of Portugal were characterized with a major volatile constituent viridiflorol [48], etc.

There is a specific problem concerning propolis volatiles coming from hives treated against *Varroa* mites. Often the treatment is performed with volatile compounds, mainly thymol. In such case, the profile of propolis essential oils is completely unnatural and is dominated up to 70 – 80% by thymol, which is only a microcomponent in untreated hives from the very same region [28].

#### *Asian propolis*

In Chinese propolis from Inner Mongolia, the major constituents of volatile oils were found to be α-bisabolol, 2-methyl-3-buten-2-ol, and 3-methyl-2-butene-1-ol [21]. High amounts of alcohols 3-methyl-3-butene-1-ol and 3-methyl-2-butene-1-ol, 4-penten-1-yl acetate and the sesquiterpene α-longipinene were also found in propolis sample of China [37]. These hemiterpene alcohols and their esters are typical poplar metabolites; they have been found also as constituents of headspace volatiles of poplar propolis from Wales [38].

Chinese researchers have applied various methods to obtain propolis volatiles prior to GC-MS analysis. In headspace volatiles (dynamic headspace sampling, DHS) of Chinese propolis from 23 regions of China, the main aroma-active components were acetic acid, 2-phenylethyl acetate and naphthalene [49]. Solid-phase micro-extraction combined with GC-MS was used for analysis of volatiles of Chinese propolis from the Beijing and Hebei provinces and again acetic acid and phenethyl acetate were among the main volatile constituents, together with phenethyl alcohol [50]. Recently, similar chemical composition, in addition to some sesquiterpenes, was found for a number of propolis samples from different regions of China [40]. Their composition was somewhat similar to the volatiles of gum from poplar trees growing in China [51]. Using microwave assisted extraction, the major compounds found in essential oils of Chinese propolis were long-chain hydrocarbons and only 17% terpenes and ester derivatives [52].

Volatile compounds of propolis sample from Turkey were analyzed by headspace-solid-phase microextraction coupled with GC/MS. Oxygenated hydrocarbons, oxygenated sesquiterpenes, aromatic alcohols and esters were the main aroma-active constituent in North Eastern Anatolian samples [41]. Distinct volatile composition was determined for propolis sample from South Eastern Anatolia (Malatya), in which monoterpenes (α-terpinene and α-terpineol) were the most abundant constituents [42].

Naik et. al. have reported chemical composition of the essential oil obtained from Indian propolis by hydrodistillation. The essential oil was shown to contain long- chain alkanes (tricosane, hexacosane, heptacosane, heneicosane), terpenoids (linalool, methyleugenol, geraniol) and phenols ((Z)-ethyl cinnamate) as major groups of compounds [27].

Outside the Temperate zone, the remarkable biodiversity of tropical flora reflects in the chemical diversity of tropical propolis constituents, including volatiles.

#### *South American propolis*

The most studied tropical propolis is Brazilian propolis, and the most popular Brazilian propolis type is the green or Alecrim propolis originating from the Asteracean shrub *Baccharis dracunculifolia *[7]. Comparative study has been carried out of green propolis and *B. dracunculifolia* volatile oils and a similarity in chemical composition (by GC-MS) detected [53,54]. Brazilian green propolis is characterized, like poplar propolis, with the predominance of sesquiterpenes. Among the major constituents, nerolidol, β-caryophyllene, spatulenol and δ-cadinene have been identified. Caryophyllene, spatulenol and δ-cadinene were the major compounds in the volatiles of several Brazilian samples from Sao Paulo, Rio de Janeiro, and Piaui States [29-31,33,55]. Green propolis from Minas Gerais State was rich in nerolidol, β-caryophyllene and selina-3,7(11) diene [32]. We found that seasonal variations in the composition of volatile oils of green Brazilian propolis are not very significant and predominantly quantitatively [29]. These results were recently confirmed by Nunes et al. using headspace GC/MS [55]. Nerolidol and the aromatic compound benzenepropanoic acid were the main aroma-active constituents in Brazilian green propolis [45]. Different sesquiterpene composition with major component longipinene was reported by Li et al. using microwave assistant extraction of volatiles from commercial Brazilian propolis [46].

In other regions of Brazil, there are propolis types with source plants other than Alecrim, so their volatile oils composition is also different. Some samples from Piaui State had monoterpenes as main constituents: α- and β-pinene, 1,8-cineole and terpinen-4-ol [33]. β-Pinene was the major constituents of samples from Rio de Janeiro State [34]. Predominance of monoterpenes α-pinene and β-pinene were also detected in the volatile oil from propolis samples collected from different regions in Brazil [35,37]. Similar volatile chemical composition was found for three propolis samples from Uruguay, one of them characterized also with high amounts of limonene [37].

Data about propolis volatile oils from other tropical regions demonstrate the chemical diversity of propolis, resulting from the specificity of the local biodiversity. For example volatile oils of Argentinean Andean propolis [39], contain high percentage of monoterpenoids, major constituents *o*-cymene and limonene. The same profile of volatile oils was found for the small shrub *Larrea nitida*, the exudates of this plant were proved to be the plant source of the particular propolis. In Yucatan, α-pinene, hexadecanoic acid and *trans-*verbenol were most abundant in propolis volatile oils [23].

#### *African propolis*

Oxygenated monoterpenes, sesquiterpenes and oxygenated aliphatic hydrocarbons were the most abundant constituents in propolis from Ethiopia [36].

In propolis volatiles from Canary Islands, sesquiterpenes (nerolidol, spatulenol, ledol) and long chain hydrocarbons were the major constituents [24].

#### *Propolis of stingless bees*

In Tropics, there are other bees, different from *A. mellifera.* They belong to the tribe Meliponini and are known as “stingless bees”. Stingless bees also collect plant resin and store it in large deposits within their nests. These deposits can be used in a similar way as honeybee propolis: in Brazil stingless bees propolis is a traditional remedy [56]. There are only a few studies of the volatile oils of stingless bees but they demonstrated that their chemical composition is different from that of honey bees from the same region, because they use different plant sources. Some of the samples studied were rich in monoterpenes, others in sesquiterpenes, and the bee species could also be of importance for the choice of plant source [22,43].

### Biological activities of propolis volatiles

Propolis plant origin is the explanation of the diverse chemical profiles of its volatile oils. It could be expected that the observed chemical differences might lead to different biological activities. The studies dedicated to the bioactivity of propolis volatiles are relatively scarce, most of them dealing with antimicrobial properties. Several authors have confirmed the activity of propolis volatiles against different microorganisms (Table 1). Among them are Gram-positive bacteria: *Staphylococcus aureus, Staphylococcus epidermidis, Micrococcus glutamicus, Bacillus subtilis, Bacillus cereus, Sarcina lutea, Streptococcus pyogenes, Streptococcus mutans, Streptococcus faecalis, *[16,20,24,26,30,35,41,57-59], but also Gram-negative bacteria such as *Escherichia coli, Enterobacter cloacae, Klebsiella pneumonie, Pseudomonas aeruginosa *[16,26,30,35,41,59]. Propolis alcohol extracts are either not active or of relatively low activity against Gram-negative bacteria; this has been confirmed by numerous authors during the last over 20 years. Propolis essential oils however demonstrated considerable activity against both Gram-positive and Gram-negative bacteria. Propolis volatiles were active also against non-pathogenic fungi and fungal human pathogens *Aspergillus niger, Saccharomyces cerevisiae, Candida albicans, Candida C. tropicalis, Candida glabrata, Cladosporium cladosporioides*, *Cladosporium sphaerospermum *[16,26,30,41] as well as against plant pathogens *Cladosporium cladosporioides* and *Cladosporium sphaerospermum *[34]. Essential oils from propolis of stingless bees also demonstrated antibacterial activity [22].

Most of the antimicrobial studies were combined with chemical analyses of the tested samples and similar activities were observed for samples with entirely different chemical characteristics. It is the combination of compounds with different chemical structure and different mechanism of action that is important for the biological activity in the case of propolis. Obviously, volatile oils also contribute to the complex way in which propolis fights the infections. A recent study demonstrated significant synergistic action between propolis alcohol extract and ginger and mint essential oils against *Staphylococcus aureus* and *Escherichia coli *[60]. Most probably a similar synergism occurs between polar and volatile constituents of propolis itself.

Only recently, some other bioactivities have been reported for propolis volatiles. Japanese researchers revealed the potential of volatiles of propolis from stingless bees to stimulate the immune system of elderly patients by increasing their natural killer cell activity in comparison to the control group [61]. Essential oils of Chinese propolis inhibited the proliferation of human colorectal cancer cells by inducing cell cycle arrest and apoptosis [62]. Propolis essential oils demonstrated therapeutic effects on anxiety of restraint-stressed mice trough antagonizing the hyperfunction of hypothalamic-pituitary-adrenal axis and improving the ability of antioxidation on brain tissue [46]. The essential oil of Indian propolis was shown to possess dose dependent repellent activity against the honeybee *Apis florea*. Such formulations might be applied by the beekeepers to keep the honeybees away from pesticide treated areas in crop fields. This would ensure the safety of honeybees and their colonies in turn [27].

The search of further bioactivities of propolis volatile oils is a promising direction in their study.

## Conclusions

It is clear that the knowledge of propolis volatile oils is far from being exhaustive. Further research is needed to reveal their chemistry and to scientifically support their medicinal properties. The most important perspectives for future research are:

– Systematic studies of volatiles of poplar type and green Brazilian propolis, to establish their typical chemical profiles for standardization purposes.

– Studies of the volatile constituents of recently discovered propolis types: red Brazilian propolis, *Clusia* type propolis from South America, Pacific propolis, etc. Their volatile constituents are almost completely unexplored.

– Studies aimed to find out if volatile propolis constituents play a role as olfactory cues for resin collection by bees.

– Studies dedicated to revealing the potential and the importance of propolis volatile oils as bioactive propolis constituents.

– It is our hope that this review would encourage some researchers to start such studies in the near future.

## Competing interests

The authors declare that they have no competing interests.

## Authors’ contributions

VB, MP and BT performed the data gathering and wrote and approved the final manuscript.
